# Medical Opinion and Movement

**Published:** 1910-02-26

**Authors:** 


### Medical Opinion and Movement.

### Gangrene after Injury to the Extremities.

Professor H. Noesske, of Kiel, writing in the *Centralblatt für Chirurgie*, describes a method of treatment he has adopted with success in cases of severe wounds of the limbs, in which cyanosis and coldness of the distal parts threatened gangrene. In such cases gangrene would appear to result from stasis of the blood. In order, therefore, to promote the circulation the author makes one or more small incisions into the cyanosed area and applies an oil dressing to prevent drying. He then places the limb in a suction apparatus at a negative pressure of 12 to 15 centimetres for eight to ten minutes. This is done two or three times a day for a week. According to the author, arterial blood exudes in increasing quantities day by day. The colour slowly improves, the sensibility of the part returns, and the wound heals satisfactorily. Professor Noesske claims to have saved portions of limbs in several cases by this method which would otherwise have been lost.

### Prophylaxis of Post-operative Peritonitis.

Pfannenstein first conceived the idea of making an injection of 10 per cent. camphorated oil into the peritoneal cavity before closing a laparotomy wound if bacterial infection was found to be present. Glimm and Hoehne, of Kiel, have carried out a series of experiments by which they have been able to show that the protective action is not due to blocking of the lymph channels by oily droplets as originally suggested, but to an irritative (chemical) peritonitis which the injection excites. The resulting exudate is strongly bactericidal and the inflammation partly occludes the channels of absorption. Hoehne finds that the injection must be made at least 24 hours before infection in order to obtain a full protective effect. In consequence of these experimental results Hoehne has treated 42 cases of laparotomy with camphorated oil injections as a prophylactic measure before operation. An account of his work appears in the *Münchener Medizinische Wochenschrift*. The cases included carcinoma uteri, pyosalpinx, ovarian cysts, etc. Under local anæsthesia a minute incision is made below the umbilicus. A blunt trocar is pushed through into the peritoneal cavity, and 30 cubic centimetres of camphorated oil, previously warmed, injected. The wound is sealed aseptically. The reaction is slight. The operation is performed 24 hours later. The peritoneum is found congested and covered with a slight fibrinous exudate. In certain cases, at any rate, such a measure might prove of value.

### Intra-thoracic Surgery.

The problem of operating upon the pleural cavity without producing pneumothorax and the resulting collapse of the lung has been to some extent solved by the various cabinets devised by Sauerbruch, Brauer, and Meyer. Such cabinets, by which the negative pressure in the pleural cavity is maintained during operation are, of course, costly and somewhat cumbersome apparatus to employ. Meltzer, working at the Rockefeller Institute, has discovered a much more simple method which promises to solve the problem in a very satisfactory manner. An account of his work appears in the *Journal of Experimental Science*. He has discovered that if a tube is introduced into a dog's trachea near to its bifurcation and a continuous stream of air is passed through the tube, the lungs are perfectly ventilated without the animal drawing breath. The principle depends upon the diffusion of air currents. The diameter of the tube should be about half of that of the trachea. The current of air passing through the tube ventilates the lung and escapes through the natural passage of the trachea. With an air pressure of about 20 mm. of mercury, obtained roughly by means of an ordinary foot-bellows, the lungs remain distended and pink, when both pleural cavities are laid wide open. The animal rests quietly and the heart beats normally, when even for hours there has been no effort at respiration. If a bottle of ether is placed in circuit with the air-tube narcosis can be obtained almost automatically, and the animal can be kept in a state of anæsthesia for three or four hours or more with little attention.

### Experimental Surgery of the Thoracic Aorta.

Alexis Carrel, also working at the Rockefeller Institute, and employing this new method of Meltzer for maintaining air-pressure in the lungs, has carried out a series of most interesting surgical experiments on the thoracic aorta in dogs. An account of the work is given in the *Journal of the American Medical Association*. He has operated on six dogs, anæsthesia being maintained in the manner already described. In three experiments the upper part of the descending aorta was cut transversely across and the ends united by a circular suture, necessitating an interruption of the circulation from 3 to 6½ minutes. These three animals recovered without incident. In the fourth experiment the ascending portion of the aorta was cut longitudinally about 3 cm. above the heart and sutured. This operation involved two interruptions in the circulation of 30 seconds each. The animal remained in good health. The fifth experiment consisted in severing the ascending aorta in the middle and interposing a segment of a large jugular vein preserved in cold storage. In the sixth experiment the upper part of the descending aorta was laid open by a longitudinal incision and a paraffined tube inserted and temporarily fastened. The anterior wall of the tubed portion was then extirpated and a segment of vena cava preserved in cold storage substituted. The tube was then taken out through a small incision and normal circulation re-established. The animal recovered, but death occurred suddenly 12 days later from hæmorrhage due to a defect in the substituted portion of vein.

From these experiments Carrel concludes that operations upon the thoracic aorta need not be dangerous, and, in fact, according to him, by this method they are as simple as abdominal operations. Suture of the descending aorta is almost identical in technique with suture of other arteries, but the ascending aorta is very friable and requires some modification of treatment. Such experimental work is highly interesting, although it is impossible at present to estimate how far these methods may be successfully applied in human surgery. If Meltzer's method of maintaining air pressure in the lungs should prove satisfactory for the human subject, it would open up a wide field for surgical skill in morbid conditions of the thorax, in which the scope of the surgeon has hitherto been so limited.

---

### Adherent Placenta.

There seems to be a distinct tendency growing up amongst one school of American obstetricians to abandon the usual Dublin or Crédé's method of accelerating the expulsion of the placenta. In *International Clinics*, Dr. Rectenwald, of Pittsburgh advocates what he calls the gravity method of dealing with abnormally adherent placentæ. In his own practice he has met with cases of this annoying complication in which he feels sure that forcible removal manually would have torn the uterus; and he has details of two cases in twelve months in his neighbourhood in which uterine rupture has thus actually been brought about. What he does and advises others to do in these cases is—after an hour or two of futile waiting and ineffectual efforts at expulsion by the usual methods—to tie a piece of gauze bandage to the cord, and to suspend by the other extremity of it a brick, which is allowed to hang over the edge of the foot of the bed. Thus steady and firm, but light, traction is continuously applied to the placenta, and in three or four days it will be found that the organ is spontaneously delivered. It is said that the patients experience no pain nor even discomfort, that no pyrexia or other evidence of septic absorption is to be feared, and that the plan is safer and sounder than the usual one of forcible removal by hand. It seems quite probable, however, that the same result would be obtained without the aid of the brick or of gravity.

---

### Leukæmic Priapism.

During the last ten years Professor Warthin has seen three cases of very prolonged and continuous priapism in leukæmic subjects. The features were in all three cases practically identical, and one of the most extraordinary was persistence post-mortem. This is ascribed to leukæmic thrombosis of the corpora cavernosa. In one case the condition persisted for fully seven months before death, and in the others for periods of lesser, but still considerable, duration. In each case there was no pain, though some difficulty in urination was experienced; sexual feeling was always entirely absent. This extraordinary symptom is apparently one of great rarity. Thus in 1907 Terrier and Dujarier could collect but 48 cases, and 16 of these were in leukæmia, which is the commonest single cause. It would also appear that priapism may be the precursor of other leukæmic symptoms, and it is said to be present in 25 per cent. of all the cases of this disease. Professor Warthin doubts the nervous hypothesis of its ætiology, preferring that of thrombosis, which explains better the persistence after death and is supported by microscopical examination of the tissues in his three cases. Obstructed venous outflow from the corpora cavernosa by a tumour or a collection of blood-clot has also been suggested as an explanation of continued priapism, and the author admits that this may account for some cases.

---

### The Dwarf Tape-worm.

The *Tænia*, or *Hymenolepis nana*, is a worm so rarely encountered in Great Britain that in most of our textbooks of medicine no mention of it is made. Nevertheless, in tropical and subtropical countries it is probably the commonest tapeworm which infests man, and it has been estimated that in Italy and Sicily 10 per cent. of the children of the poorer classes are the hosts of this parasite. Of 3,500 persons examined in tropical America for intestinal parasites, 16 were found to harbour *Hymenolepis nana*, whereas but two contained *Tænia saginata*. In Arkansas Dr. W. Deaderick estimates that the dwarf form is at least twice as common as the ordinary beef worm of our country. The length of the adult varies from 8 to 18 mm., the breadth from .33 to .58 mm., and the number of proglottides is from 100 to 200. It is thus clear that individual segments are almost indistinguishable to the naked eye. The parasites may number from a score or so up to several thousand. There is probably no intermediate host, both the cysticercoid and mature forms being possible within the human intestine. Rats and mice are believed to be sometimes the hosts of *H. nana*, but not as true intermediary hosts: the infection is probably transferred directly from patient to patient. The symptoms are much the same as those of the giant forms of tapeworm; that is to say, they are always indefinite and often absent. Diagnosis depends entirely on microscopic examination of the fæces for ova; and the best treatment is the usual male fern routine, which needs, however, as a rule to be repeated several times to effect complete cure.

---

### Submucous Resection and Septicæmia.

The popularity gained by the operation of submucous resection of the septum nasi is so widespread nowadays that the note of caution sounded by Mr. Harold Hays in the *Laryngoscope* is worthy of attention. This surgeon has encountered two cases of septicæmia following directly upon the operation, one of which had a fatal termination. In both patients, young and healthy Irish girls with marked symptoms of deflection, the septicæmia developed on the second day after an ordinary submucous resection. In the second case examination under an anæsthetic revealed a pocket of pus well above the middle turbinate bone; after this had been opened and drained the patient recovered. In the other patient a careful post-mortem examination revealed no focus of suppuration, and it seems likely that the explanation suggested may be the correct one: according to the author the venous anastomoses of the upper nasal regions offer a route by which cavernous sinus thrombosis may follow intranasal traumata even without any definite local suppurating focus. This fact, if it be one, should make everyone who proposes to undertake submucous resection, which is distinctly an extensive and mutilating operation, remember that he is dealing with a site where absolute asepsis is unattainable. The operation in question is so valuable, and has become deservedly so popular, that it is especially important to guard against any preventable complications which might tend to impair its adoption. Rhinologists will do well to bear in mind the cases which, with such commendable frankness, Mr. Hays has published.

### Post-operative Tetanus.

In a preliminary communication to the *Monthly Cyclopædia and Medical Bulletin* Dr. Rudolph Matas takes up the subject of post-operative tetanus, and endeavours to show that in some cases it has a fæcal origin, and that proper dietetic measures have an important place in prophylaxis. He says that the risk of tetanus can be absolutely eliminated in all operations upon sterile tissues in which a rigorous post-operative asepsis can be maintained until healing has occurred. But next after the exposed extremities of the limbs, which are always liable to contamination with soil containing tetanus bacilli, those regions which are liable to fæcal contamination are the most frequent sources of a tetanus infection; in this category he includes especially the anorectal region, perineum, genito-urinary tract, buttocks, groins, and thighs, and also the intestines when they have been opened in the course of any operation. Apart altogether from imperfectly sterilised catgut, or any other error in surgical technique, he claims that certain of these cases are directly due to tetanus bacilli or spores, swallowed in uncooked vegetables, berries, or fruits which have been cultivated in manured—that is, tetanised—soils. The survival of the bacilli after passage through the intestinal tract of lower animals has been abundantly proved by bacteriologists, and it is even possible that their virulence is intensified in transit. Moreover, it is also proved that 5 per cent. of healthy individuals pass stools from which tetanus bacilli can be recovered, a percentage which rises to 20 in the case of ostlers, dairymen, etc. On this is based the author's antitetanic preparation for all patients to be operated upon in regions were fæcal contamination is possible—*e.g.* for hæmorrhoids, fistula, stricture, perineoplasty, and so on. The routine is quite simple; a purge is given three days beforehand, and after this no raw or uncooked or green vegetables or fruits are allowed. In emergency cases, where this cannot be carried out, 10 c.c. of tetanus antitoxin is given subcutaneously after the anæsthetic has been administered.

### The Vision of General Paralytics.

Some conclusions of importance on this subject have been published in the *Recueil d'Ophthalmologie* by Rodiet and Pansier. Contraction of the visual fields is often an early symptom in general paralysis of the insane, and may be only for certain colours; it may also be irregular, and the colours for which the field is contracted may be from any part of the spectrum. Optic atrophy is rare: as a rule no ophthalmoscopic changes can be seen which would account for the symptoms. This well-known fact is one of the mysterious distinctions between general paralysis and tabes dorsalis which no one has yet satisfactorily explained. Scotomata, when found, are usually traceable to some other factor, not to the general paralysis. Thus alcoholism, syphilis, lead-poisoning were found by the authors in their various patients. Optic atrophy occurs in 5 per cent. or slightly more of general paralytics, but frequently some cause, such as those just mentioned, exists which might underlie it. Visual troubles in these patients are not common, and progress but slowly. Lesions of the fundus when discovered in those with visual disturbance are variable and scarcely characteristic. Optic atrophy, when present, generally appears when the disease is established and can be seen to evolve from the neuritic stage to that of atrophy.

### Adrenalin in Poisoning Cases.

It is a common and now a fairly long-established practice to combine adrenalin with cocaine and other local anæsthetics, and the practice has proved very efficacious. The rationale of this result lies, it has always been supposed, in vascular contraction and diminished (local) circulation produced by adrenalin, whereby the local anæsthetic is less quickly removed from the part to which it is applied. With the same idea of causing delayed circulation and absorption adrenalin has been lately recommended for immediate administration by the mouth in cases of poisoning. Dr. Jona has conducted some researches, of which he gives an account in the *Intercolonial Medical Journal of Australia*, to ascertain whether in fact the plan has any practical value, and he concludes that it certainly has. He has found that if adrenalin is given by the mouth soon after poison has been swallowed, and the dose is repeated after the stomach has been washed out, animals can withstand doses of poison which would otherwise cause death if the adrenalin treatment were omitted. Various poisons were used for these experiments, and the results were fairly consistent: for prussic acid, as might be expected, adrenalin does but little good unless given almost instantly, and even then it delays absorption only for a short time. There seems to be no evidence that adrenalin can act as an antidote in the strict sense of the word: the removal of the poison and the administration of the true antidote are just as necessary as ever. But practitioners will do well to bear in mind the useful hint which these researches convey, and to add a phial of adrenalin to the emergency bag. The dose may be half an ounce or more of the 1-1,000 solution, diluted with an equal quantity of water.

